# Identification of NP Protein-Specific B-Cell Epitopes for H9N2 Subtype of Avian Influenza Virus

**DOI:** 10.3390/v14061172

**Published:** 2022-05-28

**Authors:** Xiangyu Huang, Jingwen Huang, Guihu Yin, Yiqin Cai, Mengli Chen, Jianing Hu, Xiuli Feng

**Affiliations:** 1 Key Laboratory of Animal Microbiology of China’s Ministry of Agriculture, College of Veterinary Medicine, Nanjing Agricultural University, Nanjing 210095, China; 2020107044@stu.njau.edu.cn (X.H.); 2020807124@stu.njau.edu.cn (J.H.); yinguihu@stu.njau.edu.cn (G.Y.); 2021107050@stu.njau.edu.cn (Y.C.); 2020107045@stu.njau.edu.cn (M.C.); 2021207047@stu.njau.edu.cn (J.H.); 2 MOE Joint International Research Laboratory of Animal Health and Food Safety, College of Veterinary Medicine, Nanjing Agricultural University, Nanjing 210095, China

**Keywords:** H9N2 avian influenza virus, NP protein, prokaryotic expression, monoclonal antibody, antigen epitope

## Abstract

Avian Influenza (AI) caused by the H9N2 subtype of the avian influenza virus (AIV) poses a serious threat to both the poultry industry and to public health safety. NP is one of the major structural proteins in influenza viruses. B-cell determinants located on NP proteins have attracted increasing attention. In this study, based on the NP sequence of the H9N2 (A/chicken/Shandong/LY1/2017) strain, the truncated NP gene (71 AA–243 AA) was cloned and prokaryotically expressed in a pET-28a (+) vector. BALB/c mice were immunized with a purified recombinant of an NP protein to prepare a monoclonal antibody against NP proteins. The prokaryotic expression of four overlapping fragments, NP-N-96, NP-C-103, NP-C-54 and NP-C-49, were used to recognize an antigenic epitope of the NP protein. The results show that, after cell fusion, one hybridoma cell clone secreted the antibody specific to the NP protein, following screening with ELISA and indirect immunofluorescence, which is named the 4F5 monoclonal antibody (mAb). Western blotting on the overlapping fragments showed that the ^230^FQTAAQRA^237^ motif was identified as the minimal motif recognized by 4F5mAb, which was represented as the linear B-cell epitope of the NP protein. Homology analysis of this epitope shows that it was highly conserved in 18 AIVs analyzed in this study, and the epitope prediction results indicate that the epitope may be located on the surface of the NP protein. These results provide a strong experimental basis for studying the function of the NP protein of the H9N2 AIV and also strong technical support for the development of a universal assay based on an anti-NP monoclonal antibody.

## 1. Introduction

The H9N2 subtype of avian influenza was first reported in mainland China in 1992. To date, the avian influenza virus (AIV) subtype H9N2 has spread to the vast majority of mainland China and, together with subtypes H5 and H7, has become one of the predominant subtypes prevalent in poultry [1,2]. Although mainly transmitted in poultry, the avian H9N2 subtype of AIV provides the genetic fragments for co-transmitted influenza viruses through recombination. In recent years, H9N2 provides as many as seven genetic fragments except HA into other subtypes of the avian influenza genome, including H5N6, H5N2, H7N7, H10N8 and H7N9 [3,4,5,6,7]. Due to this rearrangement mechanism of H9N2, the exchange of gene fragments after its co-infection with other viruses will lead to a new genetic group of AIV with a potential pandemic, which will seriously endanger human and poultry farming.

The NP protein, encoded by the RNA of segment 5 of AIV, contains 498 amino acids in length and is one of the major structural proteins in influenza viruses [8]. The NP protein is highly conserved in all subtypes of influenza viruses and is involved in multiple stages of the viral replication cycle [9]. In the early stages of viral replication, the NP protein utilizes its nuclear localization signal to import the viral genome from the cytoplasm into the nucleus, initiating the transcription and replication of the viral genome. In the late stages of viral replication, NP exports the viral genome from the nucleus [10], and facilitates the processes of viral packaging and outgrowth release together with the M1 protein [11]. Moreover, the NP protein is a phosphorylated protein, and its phosphorylation mutants can affect the transcription and replication of the viral genome, thus inhibiting viral replication [11].

Because the H9N2 subtype of AIV has become widespread and has caused great economic losses to poultry in China [12], an effective and rapid diagnostic method for H9N2 AIV infection is urgently needed. Defined B-cell epitopes, which are recognized by specific antibodies or B-cell receptors, have become useful tools for developing diagnostic methods [13]. In this study, the NP gene was cloned and prokaryotically expressed. BALB/c mice were immunized with the recombinant NP protein to screen the monoclonal antibody (mAb) specific to the NP protein of the H9N2 subtype of AIV following hybridoma technology. A series of overlapping fragments were used to identify a linear epitope on the NP protein by Western blot analysis. This epitope was also subjected to homology analysis, as well as epitope prediction, which showed that the epitope was conserved in 18 AIV strains analyzed and was located on the surface of the NP protein. This antibody against a conserved epitope may offer valuable information for functional studies of NP proteins and for the development of new immunodiagnostic approaches.

## 2. Materials and Methods

### 2.1. Viruses, Cells and Plasmids

The H9N2 (A/chicken/Shandong/LY1/2017) strain was isolated and preserved in our laboratory [14], which was injected into a 9-day-old SPF chicken embryo allantoic cavity, and the allantoic fluid was collected and stored at −80 °C.

SP2/0 cells were cultured in RPMI-1640 medium (Vicente Biotechnology Nanjing Co., Ltd., Nanjing, China) with 20% fetal bovine serum (FBS), 1% penicillin and 100 µg/mL streptomycin at 37 °C and 5% CO_2_.

MDCK and A549 cells were cultured in RPMI-1640 medium (Vicente Biotechnology Nanjing Co., Ltd. (Nanjing, China) with 10% fetal bovine serum (FBS), 1% penicillin and 100 µg/mL streptomycin at 37 °C and 5% CO_2_.

### 2.2. NP Gene Cloning and Recombinant Protein Expression

Based on the amino acid sequences of NP gene in GenBank (No. MH018679.1), amino acid immunogenicity and conservation were analyzed with DNASTAR 11.1, and a truncated sequence with 173 amino acids (AA) (71–243 AA) was selected. The primers were designed to clone the truncated NP sequence, using the following PCR cycles: initial denaturation at 94 °C for 5 min, denaturation at 94 °C for 50 s, annealing at 56 °C for 30 s, extension at 72 °C for 30 s for 35 cycles and 72 °C extension for 5 min. The PCR product was purified by agarose gel electrophoresis and was cloned into plasmid pET-28a. The positive recombinant plasmid was identified by PCR and double digestion with *EcoR*I and *Sal*I. Then, the recombinant plasmid pET-28a-NP was transformed into an *E. coli* BL21 (DE3) strain and was induced with 100 μM IPTG for 5 h, and the supernatant and precipitate samples of the induced expressed proteins were analyzed with sodium dodecyl sulfate-polyacrylamide gel electrophoresis (SDS-PAGE) and Western blotting. Next, the recombinant NP protein was purified by nickel affinity chromatography, and the purified proteins were analyzed by SDS-PAGE.

### 2.3. Mice Immunization

Five 6~8-week-old female BALB/c mice were numbered from 1 to 5 and were immunized with the purified recombinant NP protein emulsified with Freund’s complete adjuvant at a volume ratio of 1:1. Mice were immunized by intraperitoneal injection at a dose of 100 μg per mouse. The second and third immunizations were emulsified with Freund’s incomplete adjuvant, with an interval of 2 weeks between each immunization. At one week after the third immunization, the serum was isolated from the immunized mice, and the serum antibody levels were determined by indirect ELISA. The mouse with the highest antibody level was selected for booster immunization with 200 μg of the recombinant protein.

### 2.4. Cell Fusion and Identification of Monoclonal Antibodies

At day three after the boosted immunization, the spleen cells were aseptically isolated and fused with SP2/0 cells under the action of PEG solution (P7171, SIGMA, St. Louis, MO, USA). The fused cells were cultured in a HAT medium and were screened by indirect ELISA. Positive hybridoma cells were subcloned three times to identify the specific hybridoma cell clone and were injected into BALB/c mice to prepare the ascites antibody. Then, the type and isotype of monoclonal antibodies were determined by a mouse monoclonal antibody isotype identification kit (PK20003, Proteintech, Tokyo, Japan). Finally, the specificities of monoclonal antibodies were determined by Western blotting and indirect immunofluorescence (IFA).

### 2.5. Enzyme-Linked Immunosorbent Assay (ELISA)

To screen the positive hybridoma cell clones, the ELISA diagnostic method was established using an NP recombinant protein as the encapsulated antigen. Wells of plates were coated overnight at 4 °C with 192 ng of purified NP protein diluted with carbonate-bicarbonate buffer (pH9.6) and were then blocked with 300 μL PBST (0.01 M phosphate-buffered saline (PBS), pH 7.2, 0.05% Tween 20) containing 5% skim milk at 37 °C for 1 h. After washing, 100 μL hybridoma cell supernatant was added to each well of the plate and was incubated at 37 °C for 1 h. Next, 100 μL HRP conjugated goat anti-mouse IgG (H + L) antibody at a 1:5000 dilution was added to wells for 1 h at 37 °C. After adding the chromogenic solution TMB purchased from Beyotime biotechnology Co., Ltd. (Shanghai, China) and termination solution, the absorbance was measured at 450 nm using a microplate reader (BioTek, Winooski, VT, USA).

### 2.6. NP Gene Truncation Design and Expression

To determine the B-cell epitope of this NP monoclonal antibody, the truncated NP fragment was first split into two segments: NP-N-96 and NP-C-103. The truncated NP segments were cloned by PCR with primers containing *EcoR*I and *Hind* III enzyme site, andwere cloned into a pET-28a(+) vector. After the recombinant protein was expressed, Western blotting was performed with the monoclonal antibody and His-tagged antibodies. To further localize the antigenic epitopes, the overlapping sequences of the NP protein of 165–218 AA and 194–243 AA were cloned with primers containing both *EcoR*I and *Sal*I enzyme sites, and they connected into the prokaryotic expression vector pEGX-4T-1. The recombinant plasmid was then transformed into *E. coli* BL21 (DE3) and was expressed. To finally confirm the antigenic epitopes, five polypeptides containing ten amino acids were synthesized step by step and were coupled to a BSA carrier in Shanghai Chutian Biological Co., Ltd. (Shanghai, China). The antigenic determinant of the monoclonal antibody was finally identified by Western blotting. The primers for the above NP truncated gene are shown in Table 1.

### 2.7. SDS-PAGE and Western Blotting

According to the experimental instructions, the protein samples were separated by SDS-PAGE with 12.5% and 15% and were transferred onto PVDF membranes. After blocking at room temperature for 2 h in 5% skim milk, the membranes containing protein samples were incubated overnight with a monoclonal antibody at 4 °C. After washing, the membranes were incubated with an HRP-labeled goat anti-rabbit IgG antibody (BA1050, BOSTER BIOLOGICAL CHNOLOGY) or a rabbit anti-chicken IgG/HRP (SE235, Solarbio) at 37 °C for 1 h each. Finally, the membranes were detected by a chemiluminescence detection kit (170-5061, BIO-RAD, Hercules, CA, USA).

### 2.8. Indirect Immunofluorescence Assay (IFA)

MDCK cells were infected with MOI = 0.1 H9N2 subtype AIV, and MDCK cells uninfected with viruses were used as negative controls for indirect immunofluorescence with hybridoma cell supernatants. After 24 h, the MDCK cells were fixed with 4% paraformaldehyde for 10 min and were then incubated with 0.1% Triton for 10 min. After washing, the cells were incubated with hybridoma cell supernatant overnight at 4 °C and were incubated with a fluorescent antibody (CoraLite594—conjugated goat anti-mouse IgG(H + L), SA00013-3, Proteintech) for 45 min and then with DAPI for 5 min. Finally, the cells were observed under a fluorescence microscope (Axiovert A1, Carl Zeiss AG, Jena, Germany).

### 2.9. Biological Information Analysis

A homology analysis of the obtained epitopes special to 4F5 mAb was performed using DNASTAR Megalign software (DNASTAR 11.0) to investigate the biological formations of the homology of the NP epitopes among different AIVs. Moreover, homology modeling of the NP protein of the H9N2 subtype of AIV was performed from the SWISS-MODEL website (https://swissmodel.expasy.org/, accessed on 24 March 2022). Based on the results of SWISS-MODEL modeling, the epitope positions located in the NP protein reacted to the 3D model of NP on Pymol (2.5) software (https://pymol.org, accessed on 24 March 2022) to analyze the mapped spatial characteristics as well as the biological functions of NP epitopes.

## 3. Results

### 3.1. Cloning, Expression and Purification of Recombinant NP Protein

**Figure 1:**
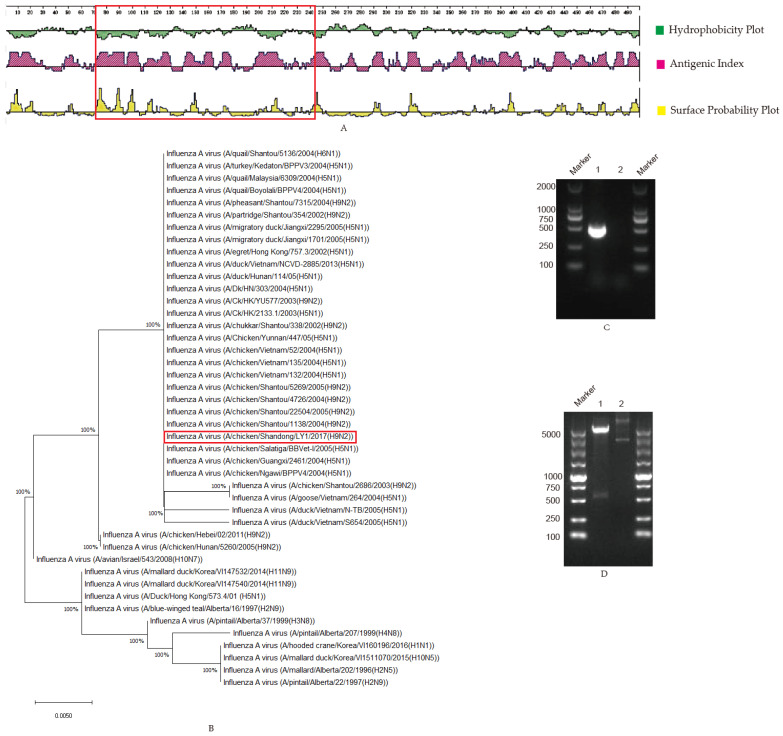

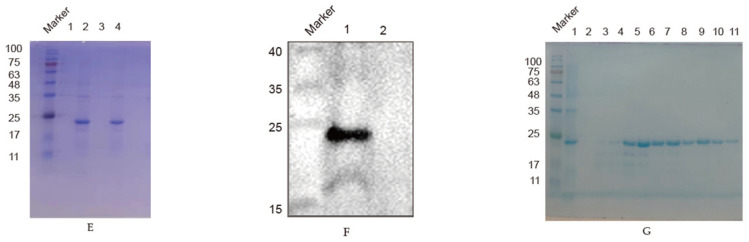
Selection and prokaryotic expression of NP truncated genes: (**A**) The structural characteristics of NP protein. Hydrophilicity, antigenicity and surface probability analysis of NP proteins were performed with the software Protean, in which the region marked in the red box was the selected fragment (71–243 AA); (**B**) Phylogenetic tree of the truncated NP protein. The homologies among the truncated NP (71–243 AA) and 44 strains of AIV were analyzed with BLASTP and software MEGA-X, and the results are shown in the phylogenetic tree. The truncated-sequence-derived strain in this study was marked in a red box; (**C**) NP PCR amplification. Lane 1 NP amplification; lane 2 negative; (**D**). Identification of recombinant plasmid pET-28a-NP by double enzyme digestion. Lane 1, pET-28a-NP; lane 2, pET-28a; (**E**) Protein expression identification. The recombinant NP protein was induced by IPTG and was analyzed on SDS-PAGE. Lane 1, pET-28a-NP not induced; lane 2, pET-28a-NP induced; lane 3, pET-28a-NP induced and supernatant after sonication; lane 4, pET-28a-NP induced and precipitated after sonication; (**F**) The recombinant protein reacted with positive serum. The fusion protein pET-28a-NP was incubated with chicken-positive serum. Lane 1 was for pET-28a-NP (24 KDa); lane 2 was for pET-28a vector control; (**G**) Purification of recombinant protein. Lane 1, effluent after column hanging; lanes 2–11, effluent after elution with different imidazole concentrations. Imidazole concentrations of 20, 30, 50, 80, 120, 150, 150, 250, 250 and 500 mM.

To investigate the structural characteristics of NP proteins [14], the antigenicity was analyzed, as shown in Figure 1A. It was observed that the region between 71 and 243 AA in the NP protein possessed an apparent antigenic index, hydrophobicity and surface probability (Figure 1A, marked by the red box), which suggests that this region in the BP protein might have good antigenic characteristics and a B-cell determinant site. In this paper, the truncated NP protein fragment with 71–243 AA was selected. The phylogenetic tree was constructed from 71 to 243 AA of NP proteins in 44 AIVs in the study (Figure 1B), and an amino acid sequence similarity analysis was performed through BLASTP (https://blast.ncbi.nlm.nih.gov/Blast.cgi, accessed on 27 October 2020) and software MEGA-X, in which the homology rate of 167 amino acid residues was up to 100% (Figure S1). The results show that the selected 71–243 AA NP protein was the relatively conserved domain among 44 AIV strains (Figure 1B and Figure S1). The truncated NP target gene was cloned with PCR, and the fragment size was 519 bp (Figure 1C), as expected. The positive recombinant plasmid pET-28a-NP was identified by double digestion (Figure 1D), and the plasmid was sequenced to prove sequence integrity in Tsingke Biotechnology Co., Ltd. (Beijing, China). The recombinant NP protein was expressed with a size of 23 KDa, which is consistent with the expected size and is expressed in inclusion bodies (Figure 1E). Moreover, it was observed that the recombinant NP protein could react specifically with positive chicken sera kept in our laboratory [15] (Figure 1F), suggesting that the expressed recombinant protein had good antigenicity. Following Ni column affinity chromatography, the recombinant NP protein showed a good purification effect at concentrations from 80 to 500 mM imidazole with the homogeneous target band (Figure 1G).

### 3.2. Screen of Monoclonal Antibody Specific to NP Proteins

**Figure 2:**
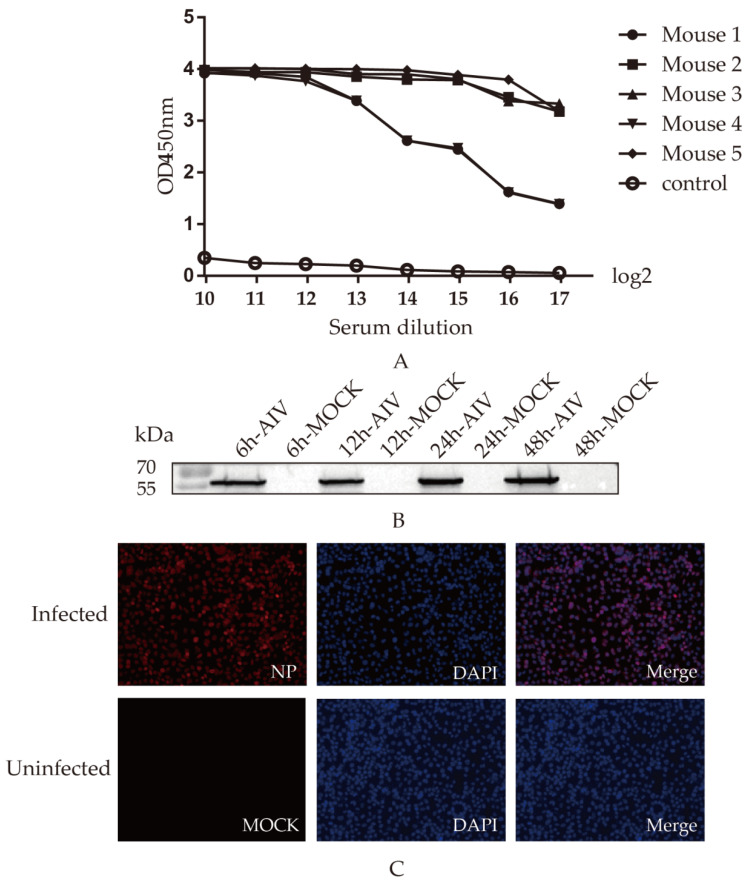
Screening and identification of monoclonal antibodies: (**A**) Antibody levels. Antibody potency in five mice one week after triple immunization. The antibody levels were presented as the absorbance value of OD450nm; (**B**) Relationship between mAb 4F5 and AIV-infected cells. A549 cells were infected with AIV, and the protein samples were collected at 6, 12, 24 and 48 h to detect recognition between NP protein and mAb 4F5 by Western blotting; (**C**) Reactivity of mAb 4F5 detected by indirect immunofluorescence. A549 cells were infected with AIV, and the reactivity of mAb 4F5 with NP protein of AIV was detected with indirect immunofluorescence. The NP protein was marked in red, and the nucleus was marked in blue.

Mice were intraperitoneally immunized three times with an interval of two weeks. At one week after the third immunization, sera were isolated from all the immunized mice, and serum antibody levels were determined by indirect ELISA, as shown in (Figure 2A), in which the No. 5 mouse with the highest level was selected to be immunized with 200 μg of recombinant protein.

At 14 d after cell fusion, the supernatant of the fused cells was detected by indirect ELISA with 2 μg/mL of coated recombinant NP protein per well, and one hybridoma cell clone was identified that secreted specific antibodies to the truncated NP protein and was named 4F5 mAb. Antibody isotype analysis showed that the isotype of 4F5 was IgG1 heavy chain, and κ was light chain (Table 2).

To determine whether the monoclonal antibody 4F5 could recognize the native NP protein, the samples collected from cells infected with H9N2 AIV were detected. The results show that 4F5 mAb could recognize the NP protein of AIV at 6, 12, 24 and 48 h from cells infected (Figure 2B). Furthermore, indirect immunofluorescence verified that 4F5 mAb could bind to cells infected with the virus (Figure 2C), suggesting that this 4F5 mAb may have the ability to recognize NP proteins.

### 3.3. Screening of B-Cell Determinants Specific to the Monoclonal Antibody

**Figure 3:**
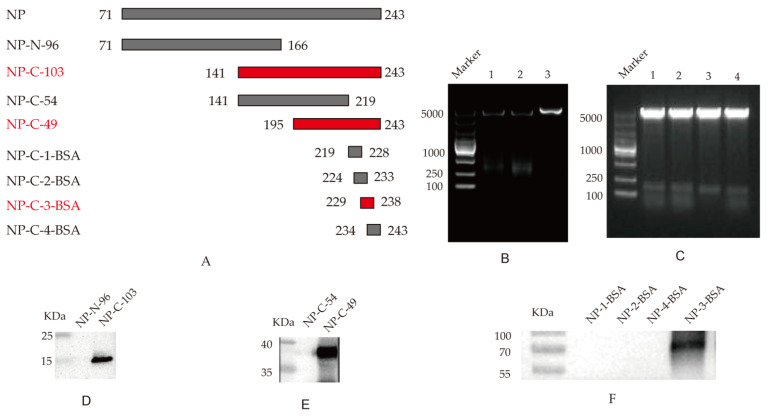
Identification of B-cell determinants special to 4F5 mAb: (**A**) Schematic diagram of NP epitope identification; (**B**) Identification of recombinant plasmids pET-28a-NP-N-96 and pET-28a-NP-C-103 by double enzyme digestion. Lanes 1, pET-28a-NP-N-96; lanes 2, pET-28a-NP-C-103; lanes 3, pET-28a vector control; (**C**) Identification of recombinant plasmids pEGX-4T-1-NP-C-54 and pEGX-4T-1-NP-C-49 by enzyme double digestion. Lanes 1–2, pET-28a-NP-C-54; lanes 3–4, pET-28a-NP-C-49; (**D**) Identification of the truncated NP-N-96 and NP-C-103 with mAb 4F5 following Western blot analysis; (**E**) Identification of the truncated NP-C-54 and NP-C-49 with mAb 4F5 following Western blot analysis; (**F**) Identification of the four coupled peptides with 4F5 mAb.

To confirm the epitopes recognized by monoclonal antibody 4F5, we constructed four truncated NP fragments and four synthetic peptides through step-by-step truncation, as shown in Figure 3A, according to the selected NP protein sequence. Firstly, two overlapping peptides spanning the truncated NP genes NP-N-96 and NP-C-103 were cloned into pET-28a (Figure 3B). After the induced expression, the specific reaction band was observed between the NP-C-103 protein and 4F5 mAb (Figure 3D), in which the AA sequences of the NP-C-103 protein were from 166 to 243 AA (Figure 3A).

To further confirm the epitope recognized by 4F5 mAb, two overlapping peptides, NP-C-54 and NP-C-49, spanning the NP genes from 141 to 243 AA, were cloned into prokaryotic expression pEGX-4T-1 vectors (Figure 3C). The Western blotting results show that the specific reaction bands were observed between the NP-C-49 protein and 4F5 mAb (Figure 3E), in which the AA sequences of the NP-C-49 protein were from 219 to 243 AA (Figure 3A).

To determine the minimal epitope of the NP protein, four peptides, ^219^YERMCNILKG^228^, ^224^NILKGKFQTA^233^, ^229^KFQTAAQRAM^238^ and ^234^AQRAMMDQVR^243^ with stepwise pentapeptides, were synthesized and coupled with BSA. The Western blotting results show a specific reaction band at peptide ^229^KFQTAAQRAM2^38^ but not with peptide ^224^NILKGKFQTA^233^ and ^234^AQRAMMDQVR^243^. It was observed that, although peptides ^224^NILKGKFQTA^233^ and ^234^AQRAMMDQVR^243^ share some common AA sequences to ^230^FQTAAQRA^237^, only ^229^KFQTAAQRAM^238^ possesses this complete AA sequence, suggesting that ^230^FQTAAQRA^237^ in the NP protein may be the minimal antigen epitope specific to 4F5 mAb (Figure 3F).

### 3.4. The Identified Epitope Is Highly Conserved in AIV Strains

**Figure 4:**
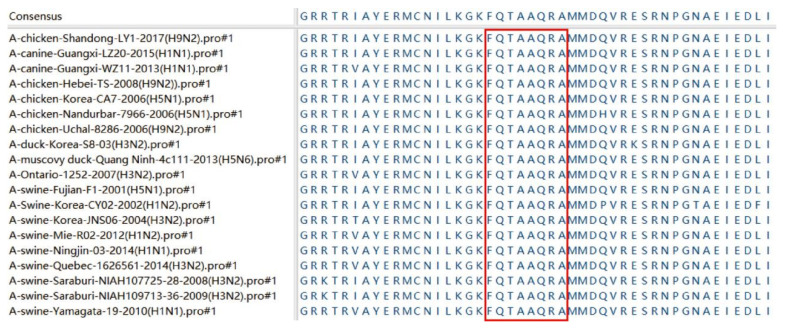
Antigenic epitope motifs were aligned with 18 AIV strains. The GenBank names of the AIV strains used are in parentheses. The homologous sequences of the identified antigenic epitopes corresponding to the different AIV strains are marked in a red box.

To compare the conservativeness of the antigen determinant, we compared the homology of the recognized epitope ^230^FQTAAQRA^237^ in the NP protein specific to 4F5 mAb with NP proteins of 18 different AIV strains, including H9N2, H1N1, H5N1, H3N2 and H1N2 subtypes. As shown in Figure 4, the comparison results show that the epitope ^230^FQTAAQRA^237^ recognized by 4F5 mAb was conserved in these 18 AIVs in this study. This result indicates that ^230^FQTAAQRA^237^ may be a relatively conserved epitope sequence in the NP protein of AIV.

### 3.5. Spatial Location Prediction of Epitope Binding

**Figure 5:**
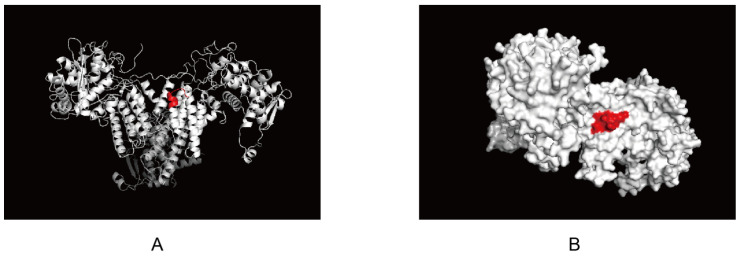
Localization of the identified epitope specific to NP protein: (**A**) Epitopes recognized by 4F5 mAb located at the junction of α-helix. This epitope was analyzed with “pymol”, which is marked in red; (**B**) Epitopes recognized by 4F5 mAb exposed on the surface of NP protein. This epitope was analyzed with Pymol. The epitope was exposed to the surface of NP protein and is marked in red.

The antigenic epitope structure of NP was analyzed using the website SWISS (https://swissmodel.expasy.org/, accessed on 24 March 2022) and the software Pymol 2.5 (https://pymol.org, accessed on 24 March 2022). The epitope recognized by 4F5 mAb, ^230^FQTAAQRA^237^, was predicted to be located at the junction of one α-helix and another α-helix (Figure 5A, marked in red). Further analysis showed that the recognized epitope, ^230^FQTAAQRA^237^, was exposed on the surface of the NP protein (Figure 5B, red marker). Combined with epitope homology analysis, this epitope may be a conserved and very important antigenic epitope in the NP protein for AIV.

## 4. Discussion

The NP protein of AIV is the internal antigenic determinant cluster of influenza viruses and is relatively conserved in different subtypes of influenza A viruses [16]. Monoclonal antibodies specific to the NP protein play an important role in the diagnosis of AIV infection, especially when new mutant AIV strains emerge and when specificity tests are not available [17].

In this paper, through the antigenic index, hydrophobicity and surface probability analysis, the region from 71 to 243 AA in the NP protein of the H9N2 subtype of AIV was selected with latent antigenic characteristics and a B-cell determinant site. Furthermore, gene sequencing and specific reactions with the positive serum to the H9N2 AIV confirmed that the recombinant truncated protein was the NP protein. These data provide an important basis for exploring the function of NP protein.

To investigate the B-cell determinant composition in the NP protein, in this paper, 4F5 mAb was obtained by the immunization of mice with recombinant truncated NP proteins, which reacted with the AIV NP protein through indirect ELISA, Western blotting, and indirect immunofluorescence methods. There are various methods to identify antigenic epitopes, including the peptide synthesis technique [18], amino acid point mutation technique [19], peptide scanning technique [20], X-ray crystallography [21], NMR technique [22], phage display technique [23], etc. In this paper, truncated-body protein expression and the peptide scanning technique were used to investigate the potential antigenic determinants in the NP protein specific to 4F5 mAb. However, only a linear epitope was screened, and conformational epitopes need to be further verified.

It was reported that various T and B-cell epitopes of NP proteins were identified. XunGui et al. reported a B-cell epitope 243–251 AA of the H1N1 influenza A virus strain A/California/04/2009 (CA/04), which was highly conserved [24]. Natalia L. et al. reported that the NP monoclonal antibody recognized four amino acid residues located at 236, 305, 372 and 470, and that the AAs at 305, 372 and 470 were highly conserved [25]. Gui-Rong B et al. established a sensitive assay using an anti-NP antibody and recognized epitopes 59–130 AA in the NP protein [26]. Moreover, the NP protein was reported to elicit a CTL response, which inhibits viral replication. McGee and Huang reported that the NP^147–155^ epitope was a T-cell epitope that could trigger an NP-specific CTL response [27]. Noisumdaeng et al. identified two conserved T-cell epitopes of NP proteins, NP^1–20^ and NP^411–430^, from the sera of recovered patients [28]. Additionally, Bethny Morrissey et al. identified four HA epitopes of the H3N2 subtype of avian influenza virus by mass spectrometry using HA monoclonal antibodies and found that an NP peptide, ^230^FQTAAQR^236^, could cross-react with HA monoclonal antibodies, in which ^230^FQTAAQR^236^ was speculated to be a possible B-cell epitope of NP [29]. In this paper, the ^230^FQTAAQR^23^^7^ was identified with a monoclonal antibody against the NP protein and was proved as the B-cell epitope of the NP protein by epitope identification. These results suggest that the identification of the epitope on the NP protein may be important, and the function of this epitope needs to be further explored.

In this paper, it was observed that this epitope was conserved in 18 AIV strains and may be exposed on the surface of the NP protein. This highly conserved and exposed epitope on the surface of the NP protein is more useful for exploring the antigen structure and function of the NP protein and provides the necessary information for redeveloping AIV immunodiagnostic techniques [29].

In conclusion, through reasonable design, the truncated NP protein was successfully expressed, and one mAb 4F5 strain was screened to the special antibody to the truncated NP protein. Furthermore, the specific B-cell epitope in the truncated NP protein to 4F5 mAb was identified through the step-by-step truncation method. These results provide a strong experimental basis for investigating the function of the NP protein of the H9N2 subtype of AIV and also lays a vital foundation for diagnosis technique development for general AIVs.

## Figures and Tables

**Table 1:** Primers of the truncated NP gene for PCR cloning.

Gene Name	Primer Name	Primer Sequence (5′–3′)	Primer Size	*T*m(°C)	Product Size (bp)
NP	NP-F	CCGGAATTCTTTGATGAAAGGAGGAACAGATACC	34	74.9	519
	NP-R	CCCAAGCTTTCGCACTTGATCCATCATTGCTCTT	34	75.1	
NP-N-96	NP-N-96-F	CCGGAATTCTTTGATGAAAGGAGGAACAG	29	71.3	288
	NP-N-96-R	ACGCGTCGACCAGAGAGCACATCCTGGGAT	30	72	
NP-C-103	NP-C-103-F	CCGGAATTCTCCAACCTAAATGATGCCAC	29	67.2	309
	NP-C-103-R	ACGCGTCGACTCGCACTTGATCCATCATTG	30	73.5	
NP-C-54	NP-C-54-F	CCGGAATTCCTGATGCAAGGATCAACTCTCCCGA	34	72.3	162
	NP-C-54-R	ACGCGTCGACATATGCAATCCTTGTTCTTCTTCCA	35	74.5	
NP-C-49	NP-C-49-F	CCGGAATTCCGGATGATAAAGCGAGGGATCAACG	34	72	147
	NP-C-49-R	ACGCGTCGACTCGCACTTGATCCATCATTGCTCTT	35	72.5	

Note: Horizontal characters are restriction endonuclease sites, as follows: GAATTC, *EcoR* I; AAGCTT, *Hind* III; GTCGAC, *Sal* I.

**Table 2:** Monoclonal antibody isotype identification and antibody potency.

Monoclonal Antibody	Antibody of Cell Culture Supernatant Potency	Ascites Potency	Heavy Chain	Light Chain
4F5	1:6400	1:128,000	IgG1	κ

## Data Availability

Not applicable.

## Supplementary Materials

The following supporting information can be downloaded at: https://www.mdpi.com/article/10.3390/v14061172/s1. Figure S1: The sequence similarities of 71 to 243 AA of NP proteins.
