# Supplementary material for: Identification of NP Protein-Specific B-Cell Epitopes for H9N2 Subtype of Avian Influenza Virus
Source: Viruses. 2022 May 28;14(6):1172. doi: 10.3390/v14061172 (PMC9228734; doi:10.3390/v14061172)
Supplement: Supplementary file 1 [file viruses-14-01172-s001.zip › viruses-1732461-supplementary.pdf]

Supplemental materials

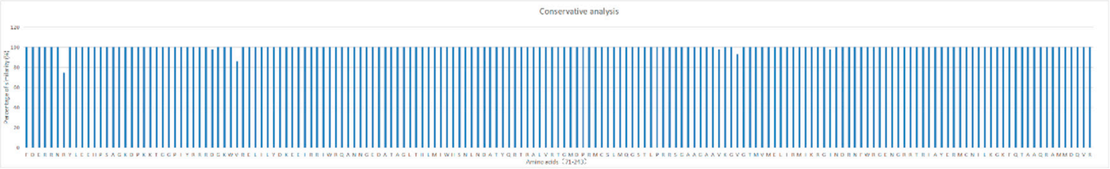

Figure S1. The sequence similarities of 71 to 243 AA of NP proteins.

Figure S1. The sequence similarities of 71 to 243 AA of NP proteins among 44 AIV strains. The similarities of each amino acid residue in the selected NP protein was analyzed through BLASTP, and was presented as the histogram.
